# Secondary Traumatization in Caregivers Working With Women and Children Who Suffered Extreme Violence by the “Islamic State”

**DOI:** 10.3389/fpsyt.2018.00234

**Published:** 2018-06-05

**Authors:** Jana K. Denkinger, Petra Windthorst, Caroline Rometsch-Ogioun El Sount, Michael Blume, Hes Sedik, Jan I. Kizilhan, Niamh Gibbons, Phuong Pham, Jennifer Hillebrecht, Nora Ateia, Christoph Nikendei, Stephan Zipfel, Florian Junne

**Affiliations:** ^1^Department of Psychosomatic Medicine and Psychotherapy, University Hospital Tübingen, Tübingen, Germany; ^2^Department of Non-Christian Religions, Values, Minorities and Northern Iraq Projects, Ministry of State of Baden-Württemberg, Stuttgart, Germany; ^3^Department of Mental Health and Addiction, Baden-Württemberg Cooperative State University Villingen-Schwenningen, Villingen-Schwenningen, Germany; ^4^Harvard Humanitarian Initiative, Harvard University, Cambridge, MA, United States; ^5^Rehabilitation Psychology and Psychotherapy, Department of Psychology, University of Freiburg, Freiburg, Germany; ^6^Department of Child and Adolescence Psychiatry and Psychotherapy, University Hospital Tübingen, Tübingen, Germany; ^7^Department of General Internal Medicine and Psychosomatics, University Hospital Heidelberg, Heidelberg, Germany

**Keywords:** secondary traumatization, refugees, caregiver, trauma, resilience, attachment style, genocide, Yazidi

## Abstract

**Introduction:** Refugees fleeing persecution, torture, or sexual violence are at high risk of developing both acute and chronic psychological disorders. Systematic violence, as committed against the Yazidi minority in Northern Iraq by the terror organization known as the Islamic State (IS), can be seen as a particularly traumatic burden to the victims, but also to caregivers providing treatments and assistance to them. The intense exposure to traumatic content may cause secondary traumatization in respective caregivers. This study aims (1) to identify the prevalence of secondary traumatization in caregivers working with traumatized women and children from Northern Iraq; (2) to determine the specific distressing factors and resources of the caregivers; as well as (3) to analyze whether caregivers' personal history of trauma or flight, attachment styles, working arrangements as well as support offers qualify as risk or resilience factors for secondary traumatization.

**Materials and Methods:** In this cross-sectional study, *N* = 84 caregivers (social workers, psychotherapists/physicians, and interpreters) in the context of a Humanitarian Admission Program (HAP) for women and children traumatized by the so called IS were investigated about their work-related burdens and resources. Secondary traumatization was assessed with the Questionnaire for Secondary Traumatization (FST). To identify relevant determinants for secondary traumatization multiple linear regression analyses were performed.

**Results:** Secondary traumatization was present in 22.9% of the participating caregivers, with 8.6% showing a severe symptom load. A personal history of traumatic experiences, a personal history of flight, a higher number of hours per week working in direct contact with refugees as well as a preoccupied attachment style were detected as risk factors for secondary traumatization. A secure attachment style could be identified as a resilience factor for secondary traumatization.

**Discussion:** Caregivers working with traumatized refugees are at high risk of developing secondary traumatization. Based on the findings of this study and theoretical considerations, a framework of classification for different types of trauma-associated psychological burdens of caregivers working with traumatized refugees is proposed. Implications for the training and supervision of professionals in refugee- and trauma-care are discussed.

## Introduction

In 2017, the UN Refugee Agency (UNHCR) registered an unprecedented number of 65.6 million forcibly displaced people worldwide. Among them, 22.5 million people are classified as refugees, defined as people who have been forced to flee their home country because of persecution, war, or violence (1). Germany received 222.683 applications for asylum in 2017, out of which 198.317 were first-time applications (2). In 2016, Germany registered the highest number of asylum applications in the last decade: 722.370 fist-time applications for asylum in Germany. In both years, around half of refugees trace their origin to one of three countries: Syria, Afghanistan, or Iraq (2).

Although the prevalence rates of mental disorders in refugees worldwide varies widely among different studies, a large meta-analytic review recently identified prevalence rates of 30.6% for post-traumatic stress disorder (PTSD) and rates of 30.8% for depressive disorder among refugees (3, 4). In this context and in general, man-made traumas such as torture and especially sexual violence are a strong predictor for developing PTSD (5, 6) and comorbid depression (7).

Religious minorities from Northern Iraq, such as the Yazidis, are heavily burdened victims of man-made traumas due to the extreme violence and terror of the so-called Islamic State (IS). The jihadist terror organization is also known as the Islamic State in Iraq and Syria (ISIS), the Islamic State in Iraq and the Levant (ISIL), or as Daesh, the acronym of the group's previous Arabic name. In August 2014, IS attacked and subjugated the Yazidis and other religious minorities in the area of Mount Sinjar in Nineveh governorate, Iraq. In a retrospective household survey, it was estimated that 2.5% of the Yazidi population was either killed or kidnapped during these attacks (8). Men, women, and children were exposed to extremely brutal violence including forced religious conversion, enslavement, extreme torture, and execution. Furthermore, women and girls were exposed to systematic sexual violence by IS-fighters whereas boys were trained to become child soldiers (8, 9). Approximately half of the victims who were able to flee from IS show full syndrome of PTSD [42.9%, (10)]. Furthermore, 39.5% of these victims suffer from major depression (10). In the population of IS-victims, women are more likely than men to suffer from PTSD and major depression (10). The United Nations declared the crimes against the Yazidis as an ongoing genocide (11).

The efficacy of trauma-specific treatments for refugees in Germany is documented and its importance is well-grounded (12, 13). Nevertheless, caregivers working with traumatized refugees are confronted with multiple challenges and burdens in their everyday work. Due to their close contact with their clients, caregivers are frequently exposed to details of extremely traumatic events (14). The repeated exposure to their clients' traumatic memories can lead to the transference of typical trauma symptoms such as hyperarousal, avoidance, and intrusions to the caregivers even if they were never exposed to the traumatic events themselves (15). This phenomenon is called secondary traumatization and can be identified in various helping professions including social workers and trauma therapists (16–18). In addition, Kindermann et al. recently reported that 21% of interpreters working with refugees suffered from secondary traumatization (19). The general prevalence rates of secondary traumatization in caregivers illustrate the need for research on risk and resilience factors of secondary traumatization especially in professionals working with highly traumatized patients. With regards to specific predictors of secondary traumatization, one factor that has been controversially discussed in the previous literature is a personal trauma history of the caregiver (20–22). In the context of refugee-care, especially an own history of flight could be particularly relevant. Another factor commonly found to be associated with secondary traumatization is the degree of exposure (23). Furthermore, there is some evidence that supervision can be protective to harmful changes in professionals' views of themselves, others, and the world, as a result of exposure to traumatic details of their client's history (24). Moreover, peer-to-peer consultation (intervision) is often recommended when working with trauma-patients to prevent work related stress (25).

Previous studies found an association between attachment styles and vulnerability for mental disorders, inter alia PTSD (26). Attachment theorists propose that early childhood experiences lead adults to exhibit distinct attachment patterns which can be characterized as secure or insecure attachment styles. Insecure attachment can be divided into preoccupied, fearful, or dismissing attachment styles (27). Several studies indicate that a secure attachment style may have a protective effect on the development of PTSD whereas insecure attachment styles are associated with higher levels of PTSD symptoms (26, 28). So far, there has been preliminary evidence that attachment styles may also have an impact on secondary traumatization (19, 29). In a survey with interpreters, Kindermann et al. found that a dismissing attachment style serves as a risk factor and a secure as well as a preoccupied attachment style qualify as resilience factors for secondary traumatization (19).

However, there is little research about the burden of secondary traumatization and the relevance of different risk and resilience factors in caregivers so far. To our knowledge, data on secondary traumatization in professionals working specifically with IS-victims who have suffered extreme mental and physical torture and the most brutal forms of sexual violence are also missing. Such extreme trauma contents may pose an increased risk for secondary traumatization in caregivers.

This is the first study to investigate the prevalence and determinants of secondary traumatization in caregivers as well as distressing factors, resources, and needs in their everyday work with IS-traumatized refugees. The aims of this study are to (1) identify the prevalence of secondary traumatization in caregivers working with IS-traumatized refugees, (2) to analyze the caregivers' specific distressing factors and resources and (3) to determine risk and resilience factors of secondary traumatization in caregivers by testing the hypothesis that supervision, intervision, secure, and preoccupied attachment styles serve as resilience factors of secondary traumatization whereas an own trauma history, own experiences of flight, more hours per week of direct contact with refugees as well as fearful and dismissing attachment styles serve as risk factors for secondary traumatization.

## Methods

### Study design and ethical considerations

This cross-sectional study used validated quantitative psychometric survey instruments as well as self-developed quantitative questionnaire-items. The study was approved by the ethics board of the Medical University of Tübingen (ethics application No. 189/2017BO2).

### Baden-württemberg humanitarian admission program

To ensure a safe environment and adequate medical and psychological treatment to the survivors of the Yazidi genocide from 2014, the Ministry of State of Baden-Württemberg, Germany, implemented a Special Quota Project known as the Baden-Württemberg Humanitarian Admission Program (HAP). In 2015/2016, the HAP offered 1,100 especially vulnerable women and children who survived IS-violence the opportunity to migrate to Germany and receive medical and psychological treatment as well as housing and education with the aim of enabling long-term integration into the German society (30–32). The women and children of the HAP are now living in 22 districts in Germany, where an interprofessional team of caregivers including social workers, psychotherapists, doctors, and interpreters is involved in the provision of specialized and target-group adjusted services (31). Currently, Germany has the largest Yazidi diaspora worldwide with a strong sense of community identification (33).

### Sample

Initially all registered caregivers of the HAP were invited to a HAP-networking meeting. With the invitation, the caregivers received the study information and were invited to participate. At the networking meeting on April, 27, 2017, all interested caregivers answered the below described questionnaires before the program of the networking meeting started (to avoid contamination and cross-influence of participants). Participation was voluntary, pseudonymized, and conducted in German language.

Out of 132 registered caregivers of the HAP, *N* = 96 participants took part in the study (response rate = 72.7%). Since secondary traumatization is only relevant for caregivers working in direct contact with traumatized patients, we excluded 12 participants who were exclusively working administratively in the project. In the end, a final sample of 84 caregivers was included in the analysis. Sociodemographic characteristics of the sample are depicted in Table 1.

**Table 1:** Sample description of participating caregivers of the Baden-Württemberg Humanitarian Admission Program (*N* = 84).

	***M* (*SD*, range)**
Age (years)	44.0 (13.0, 23–66)
Work experience (months)	
in the HAP	16.3 (6.7, 1–26)
with traumatized patients	72.1 (93.7, 1–360)
***N* (%)**	
**Gender**	
Female	78 (94.0)
Male	5 (6.0)
**Profession**	
Social workers	50 (59.5)
Interpreters	11 (13.1)
Health care professionals (psychologists, psychotherapists, physicians)	10 (11.9)
Creative therapists	6 (7.1)
Administrators with direct contact to beneficiaries	4 (4.8)
Others	3 (3.6)
Further qualifications for working with refugees	36 (45.0)
Trauma relevant qualification	16 (19.0)
**Voluntary/professional basis**	
Professional	73 (88.0)
Voluntary (within own profession)	2 (2.4)
Voluntary (outside of own profession)	8 (9.6)
**Organization of help offers**	
Working with children	11 (13.4)
Working with adults	6 (7.3)
Working with adults and children	65 (79.3)

### Survey instruments

Sociodemographic data and context characteristics regarding the work in the HAP were assessed via self-developed questionnaire items. In addition, we asked the caregivers if they have experienced traumatic situations (e.g., violence, accidents) themselves and if they had a personal flight history due to war or conflicts. Furthermore, two standardized scales were adapted to assess secondary traumatization and attachment styles and two self-developed item sets were used to capture distressing factors and resources of the caregivers.

The professional lifetime version of the Questionnaire of Secondary Traumatization [FST; (17, 34)] was used to assess the severity of secondary traumatization. We chose the professional lifetime version of the FST over the acute version to capture secondary traumatization during the most stressful time period in the HAP. Expert-interviews we conducted with HAP-professionals for preparing the study revealed that the time period after the women arrived were especially stressful for the caregivers. The FST consists of five subscales: “Intrusion,” “avoidance,” “hyperarousal,” “parapsychotic sense of threat,” and “PTSD-comorbidities.” Participants are prompted to rate how often these symptoms occurred during the first week of the time period with the highest level of distress in the professional context in question (here: refugee care). The FST sum scores can yield sum scores from 31 to 155. Participants scoring under 65 show no signs of secondary traumatization, sum scores above 65 indicate clinical relevant symptoms of secondary traumatization. Between 65 and 82, sum scores classify moderate secondary traumatization, while scores above 82 refer to severe secondary traumatization (17). The FST shows a high internal consistency with Cronbach's α = 0.94 (34).

The Relationship Questionnaire (27) was used in the German Version to assess adult attachment styles. On a 7-point Likert scale, four attachment patterns (secure, dismissing, preoccupied, and fearful), defined by using a combination of a person's self-image and image of others (positive or negative), can be rated. A secure attachment style indicates positive internal models of the self and others. High scores on the dismissing attachment style represent a positive evaluation of the self and a negative disposition toward other people. A preoccupied attachment style corresponds to a negative model of the self and a positive model of others. Scoring high on the fearful subscale refers to negative models of the self and others. An international, large-scaled study could identify the following mean values (35): Secure: *M* = 4.3 (*SD* = 1.7), dismissing: *M* = 3.7 (*SD* = 1.8), preoccupied: *M* = 3.4 (*SD* = 1.9), and fearful: *M* = 3.5 (*SD* = 2.0).

Since the distressing factors and resources of the HAP-caregivers are highly context specific, two self-constructed 7-point Likert scaled item sets were applied. We asked the participants to rate from 1 (very low) to 7 (very high) how burdened/supported they feel due to the distressing/helpful factors depicted in Tables 2, 3. The item set measuring distressing factors consisted of 16 items with a high internal consistency of Cronbach's α = 0.86. The nine items of the item set assessing different resources are more heterogeneous with a sufficient internal consistency of Cronbach's α = 0.61. The questionnaire-items were developed by the research team consisting of psychotherapists, psychologists, and physicians and piloted as well as adapted by means of expert-interviews using the think aloud method (36) with professionals (psychotherapists, social workers, and interpreters) working in the HAP context.

**Table 2:** Self-constructed questionnaire items assessing distressing factors of caregivers working with IS-traumatized refugees.

**Questionnaire-Items: Distressing factors**
“*Autonomy/dependency conflicts between beneficiaries and caregivers i.e., beneficiaries either show themselves as overly dependent or overly autonomous in the relationship with the caregiver”* “*Beneficiaries' contacting of offenders in Iraq”* “*Beneficiaries' decision to return to Northern Iraq”* “*Excessive expectations of beneficiaries regarding the caregivers' profession/role”* “*Pain symptoms of beneficiaries”* “*Reports on beneficiaries' traumatic experiences”* “*Subjective lack of appreciation from beneficiaries”* “*Witnessing the suffering of beneficiaries”* “*Cultural differences in general between beneficiaries and caregivers”* “*Different attitudes toward (physical) closeness and distance between beneficiaries and caregivers”* “*Differences in child-rearing between beneficiaries and caregivers”* “*Differences regarding gender roles between beneficiaries and caregivers”* “*Differences regarding marriages between beneficiaries and caregivers”* “*Religious differences between beneficiaries and caregivers”* “*Intermixture of private and professional aspects of the caregiver”* “*Humanitarian developments in Northern Iraq e.g., exhumations of Yazidi mass graves by the United Nations to enable certainty about the loss of dead family members or prosecution of responsible fighters”*

**Table 3:** Self-constructed questionnaire items assessing supportive factors of caregivers working with IS-traumatized refugees.

**Questionnaire-Items: Supportive factors**
“*Appreciation from beneficiaries”* “*Appreciation of work from society”* “*Communication with colleagues from other care centers”* “*Communication with colleagues from own care centers”* “*Supervision”* “*Support from security services”* “*Support from superiors”* “*Already acquired knowledge and competence”* “*Awareness of doing something meaningful”*

### Statistical analysis

For sample description, means, percentages, and distributions are reported. For the analysis of differences in means, Mann-Whitney-U tests for independent samples were applied since the data was not normally distributed. To test correlations of determinants with the respective outcome (FST-scores), Spearman rho test was applied as a non-parametric measure. Multiple linear regression analyses were performed to assess whether determinants were associated with FST-scores. Due to the small sample size, we conducted two multiple linear regression analyses and separated the potential determinants with regards to content in terms of our hypothesis to a regression model with potential risk factors and one model with potential resilience factors for secondary traumatization. The level of significance for all analyses was set at α = 0.05. All statistical analyses were performed using IBM SPSS Statistics version 24 (37).

## Results

### Prevalence of secondary traumatization

**Figure 1:**
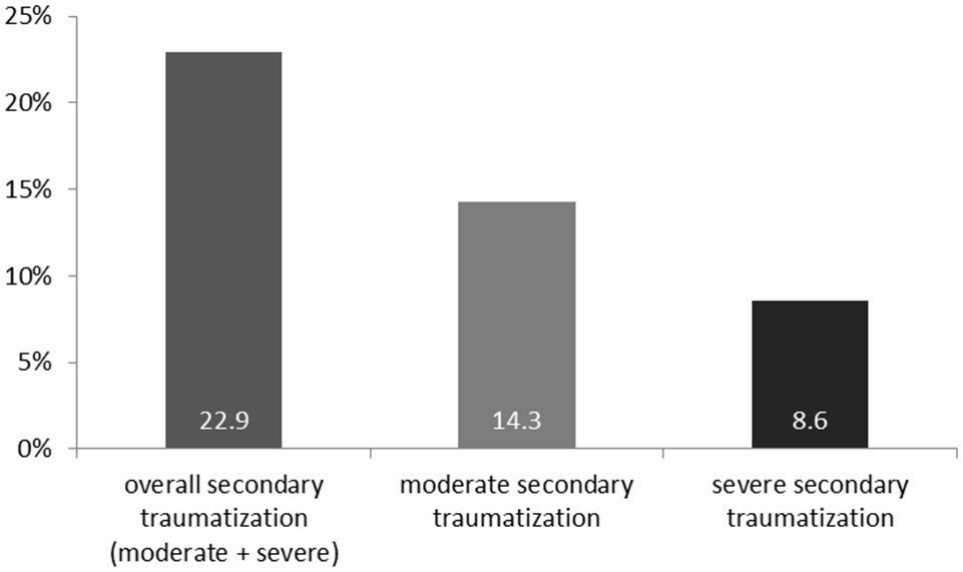
Percentages of caregivers with secondary traumatization measured by the Questionnaire of Secondary Traumatization (FST).

Applying the FST-diagnostic criteria, secondary traumatization was present in 22.9% of the participating caregivers at least once throughout their work as a refugee-caregiver, with 14.3% indicating a moderate secondary traumatization and 8.6% showing severe secondary traumatization (Figure 1). The mean FST-score was *M* = 52.94 (*SD* = 16.92). Conspicuously, 62.5% of interpreters show secondary traumatization. FST-scores for the whole sample and for the main professions individually are presented in Table 4.

**Table 4:** Secondary traumatization of caregivers working with IS-traumatized refugees in categories for the whole sample and the three main professions individually.

**FST-categories**	***N* (%)**
**WHOLE SAMPLE (*****N*** = **70)**	
No ST	54 (77.1)
Moderate ST	10 (14.3)
Severe ST	6 (8.6)
**SOCIAL WORKERS (*****N*** = **42)**	
No ST	34 (81.0)
Moderate ST	6 (14.3)
Severe ST	2 (4.8)
**HEALTH CARE PROFESSIONALS (*****N*** = **9)**	
No ST	8 (88.9)
Moderate ST	1 (11.1)
Severe ST	0 (0)
**INTERPRETERS (*****N*** = **8)**	
No ST	3 (37.5)
Moderate ST	1 (12.5)
Severe ST	4 (50.0)

*ST, secondary traumatization; FST, Questionnaire for Secondary Traumatization*.

### Workload

Caregivers in the HAP work on average *M* = 17.78 (*SD* = 14.12) hours per week while they spend *M* = 11.84 (*SD* = 10.88) hours in direct contact with the beneficiaries. Furthermore, HAP-caregivers care for *M* = 19.49 women (*SD* = 27.58) and *M* = 23.19 (*SD* = 24.16) children.

### Personal trauma and flight history of caregivers

A personal trauma-history was reported by 24.1% of caregivers. Examining each profession individually, 40.0% of all participating creative therapists, 27.3% of interpreters, 23.4% of social workers, and 22.2% of health care professionals (psychologists, psychotherapists, and physicians) had experienced at least one traumatic event in their life.

A personal history of flight was reported by 7.6% of caregivers. More precisely, almost half of the interpreters (45.5%) and 10.0% of health care professionals reported a personal history of flight.

### Attachment styles (RQ)

With a mean of *M* = 5.77 (*SD* = 1.03) a secure attachment style is predominant in HAP-caregivers. The ratings to each attachment style are presented in Table 5.

**Table 5:** Attachment styles of the caregivers of the Baden-Württemberg Humanitarian Admission Program.

**Attachment styles**	***M***	***SD***	**Min**	**Max**
Secure attachment style	5.77	1.03	3	7
Dismissing attachment style	3.81	1.73	1	7
Preoccupied attachment style	2.05	1.29	1	7
Fearful attachment style	1.94	1.17	1	6

*Relationship Questionnaire Ratings are defined from 1 (“doesn't describe me”) to 7 (“very accurately describes me”)*.

### Burdens of caregivers

**Figure 2:**
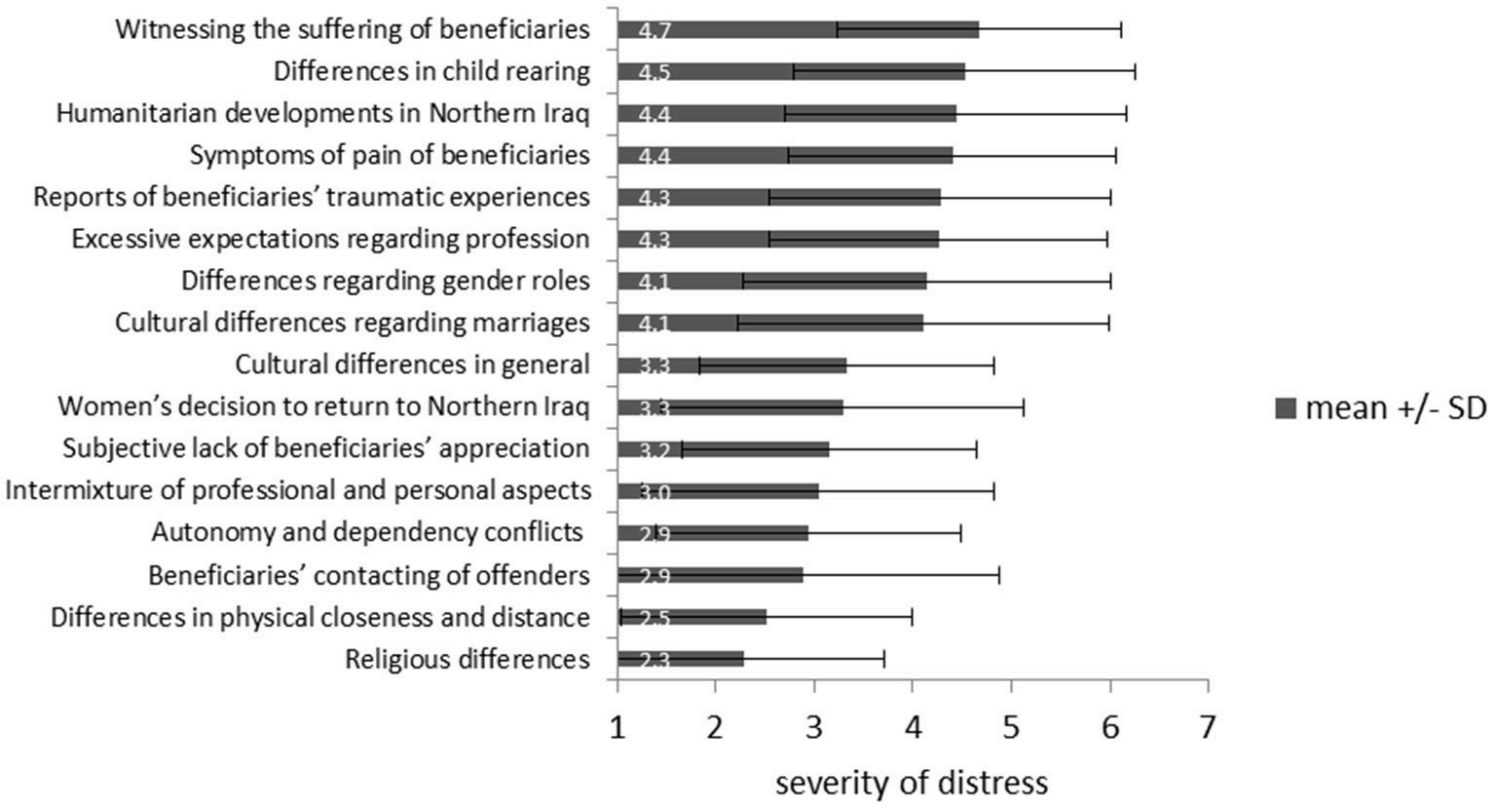
Ranking of distressing factors of caregivers working with IS-victims from 1 (“very low distressing”) to 7 (“very high distressing”).

Based on the list of the self-developed questionnaire-items regarding distressing factors in the HAP, the caregivers rated “*witnessing the suffering of beneficiaries”* (*M* = 4.67, *SD* = 1.44), “*differences in child-rearing”* (*M* = 4.53, *SD* = 1.73), and “*humanitarian developments in Northern Iraq”* (*M* = 4.44, *SD* = 1.78) as the most distressing factors in their work (Figure 2).

### Resources of caregivers

**Figure 3:**
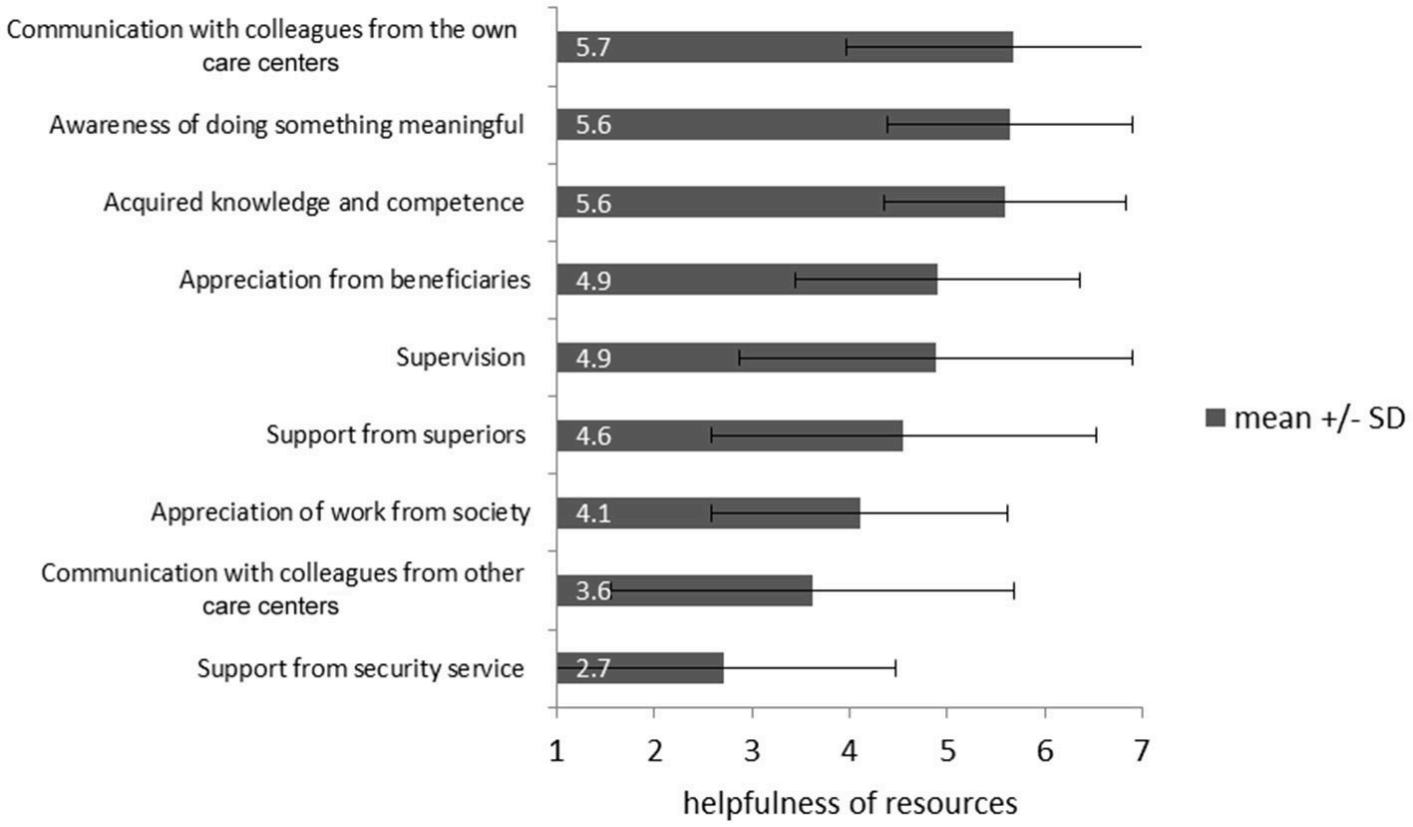
Ranking of resources of caregivers working with IS-victims from 1 (“not helpful”) to 7 (“very helpful”).

The caregivers considered “communication with colleagues from own care center” (*M* = 5.67, *SD* = 1.71), “awareness of doing something meaningful” (*M* = 5.64, *SD* = 1.25), and the “already acquired knowledge and competence” (*M* = 5.59, *SD* = 1.24) as the most helpful work-related resources (Figure 3).

### Perceived need for supervision

While 64.1% of the caregivers stated that they had the opportunity for supervision and 61.5% for intervision, 71.6% of the participating caregivers rated their need for supervision as “rather high” to “very high” (*M* = 5.04, *SD* = 1.68) on a scale from 1 = “very low” to 7 = “very high.” The wish for case-related supervision was “rather high” to “very high” in 82.6% of caregivers (*M* = 5.51, *SD* = 1.45), whereas 57.6% wished for supervision related to their personal burdens in a “rather high” to “very high” way (*M* = 4.7, *SD* = 1.87). Specifically, 40.1% of caregivers rated their wish to have individual supervision as “rather high” to “very high” (*M* = 4.04, *SD* = 1.89) and 67.5% rated their wish for group supervision as “rather high” to “very high” (*M* = 4.90, *SD* = 1.89).

### Associations with secondary traumatization

Caregivers with own traumatic experiences (Median = 56.0) showed significantly stronger symptoms of secondary traumatization than caregivers without own experiences of trauma (Median = 50.0), *U* = 240.5, *p* = 0.008. However, no significant differences in severity of secondary traumatization emerged between caregivers with and without intervision, supervision, relevant specific trainings, or whether a caregiver worked on a volunteer or a professional basis. Associations between individual and environmental characteristics and FST-scores are presented in Table 6.

**Table 6:** Associations between secondary traumatization symptom load (FST-scores) and sociodemographic factors, context characteristic, workload, attachment styles, distressing factors, and resources of caregivers working with IS-traumatized refugees.

	**FST-scores**	
	***r_*s*_***	***p***
**SOCIODEMOGRAPHIC FACTORS**		
Age	−0.09	0.487
Work-experience with traumatized patients	0.02	0.887
Work experience in the HAP	0.18	0.134
**WORKLOAD**		
Working hours per week in the HAP	0.34	0.006^**^
Working hours per week in direct contact with beneficiaries	0.33	0.006^**^
Number of women caring for	0.17	0.173
Number of children caring for	0.27	0.030^*^
**ATTACHMENT STYLES**		
Secure attachment style	−0.26	0.034^*^
Dismissing attachment style	0.20	0.106
Preoccupied attachment style	0.29	0.015^*^
Fearful attachment style	0.15	0.221
**DISTRESSING FACTORS**		
“*Autonomy/dependency conflicts between beneficiaries and caregivers”*	0.31	0.009^**^
“*Reports on beneficiaries' traumatic experiences”*	0.39	0.001^**^
“*Witnessing the suffering of beneficiaries”*	0.43	< 0.001^***^
“*Beneficiaries contacting of offenders in Northern Iraq”*	0.36	0.004^**^
“*differences regarding gender roles between beneficiaries and caregivers”*	0.26	0.034^*^
“*intermixture of private and professional aspects of the caregiver”*	0.49	< 0.001^***^
“*excessive expectations of beneficiaries regarding the caregiver's profession/role”*	0.32	0.007^**^
**RESOURCES**		
“*Communication with colleagues from own care center”*	−0.28	0.019^*^
“*Communication with colleagues from other care centers”*	−0.38	0.002^**^

*Only significant associations between distressing factors and resources and FST-scores are depicted in this table. HAP, Baden-Württemberg Humanitarian Admission Program, FST, Questionnaire of Secondary Traumatization*,

* *p < 0.05*,

** *p < 0.01*,

*** *p < 0.001*.

### Potential risk factors for secondary traumatization

Multiple linear regression analysis was performed to assess to what extent potential risk factors explain the variance of FST-scores in caregivers. To identify risk factors for secondary traumatization, we included variables in the regression model which could be identified as risk factors for secondary traumatization for other samples before (e.g., interpreters) or have been discussed in recent literature to put an individual at risk for secondary traumatization such as personal trauma history, specific attachment styles as well as degree (duration) of exposure (15, 19, 23).

The data met all the assumptions for multiple regression analysis (Durbin-Watson-Statistic = 1.96). A significant regression equation was found [*F*_(5, 57)_ = 4.541, *p* = 0.001], with *R*^2^ = 0.285. The result of the regression indicates that the identified risk factors explain 28.5% of the variance of secondary traumatic symptom load. Comparing the individual contributions of variances for single factors, standardized beta values are highest for personal history of flight, hours per week working in direct contact with HAP beneficiaries and personal history of traumatic experiences (Table 7).

**Table 7:** Linear regression model for potential risk factors for secondary traumatization in caregivers.

**Variables**	***N***	***b* (95% CI)**	***SE b***	**β**	***p***	**Tolerance**
Constant		36.38 (25.93, 46.83)	5.22		0.000^***^	
Personal history of traumatic experiences	63	11.44 (2.64, 20.24)	4.40	0.305	0.012^*^	0.916
Personal history of flight	63	21.71 (6.57, 36.86)	7.56	0.331	0.006^**^	0.944
Hours per week working in direct contact with beneficiaries	63	0.45 (0.11, 0.79)	0.17	0.311	0.012^*^	0.882
Dismissing attachment style	63	0.35 (−1.86, 2.56)	1.10	0.038	0.754	0.861
Fearful attachment style	63	2.71 (−0.36, 5.78)	1.53	0.208	0.083	0.905

*R^2^ = 0.285, Adjusted R^2^ = 0.222, β = standardized beta*,

* *p < 0.05*,

** *p < 0.01*,

*** *p < 0.001*.

### Potential resilience factors for secondary traumatization

A second multiple linear regression analysis was calculated to assess to what extent potential resilience factors for caregivers working with traumatized refugees can explain variance of severity of secondary traumatization. We used secure and preoccupied attachment styles as independent variables, because Kindermann et al. showed that those two attachment styles serve as resilience factors for secondary traumatization in interpreters working in refugee-care (19). In addition, we added supervision and intervision as independent variables to the model, since their positive effect on caregivers has been mentioned in previous literature (38, 39).

The data met all the assumptions for multiple regression analysis (Durbin-Watson-Statistic = 1.99). A significant regression equation was found [*F*_(4, 60)_ = 2.995, *p* = 0.025], with *R*^2^ = 0.166. The results of the regression indicate that the regression model explains 16.6% of the variance of secondary traumatization symptom load. Comparing the individual contributions of variances for single factors, standardized beta values were highest for secure attachment style and preoccupied attachment style. However, whereas secure attachment style is associated negatively with FST-scores, a preoccupied attachment style is associated with FST-scores in a positive way. Supervision and intervision do not contribute significantly to explaining the variance in the FST-scores (Table 8).

**Table 8:** Linear regression model for potential resilience factors for secondary traumatization in caregivers.

	***N***	***b* (95% CI)**	***SE* b**	**β**	***P***	**Tolerance**
Constant		70.28 (46.55, 94.01)	11.87		0.000^***^	
Secure attachment style	65	−4.33 (−8.10, −0.56)	1.89	−0.274	0.025^*^	0.975
Preoccupied attachment style	65	3.22 (0.35, 6.09)	1.44	0.273	0.028^*^	0.936
Supervision	65	0.54 (−7.52, 8.60)	4.03	0.016	0.893	0.937
Intervision	65	0.33 (−7.59, 8.25)	3.96	0.010	0.933	0.942

*R^2^ = 0.166, Adjusted R^2^ = 0.111, β = standardized beta*,

* *p < 0.05*,

*** *p < 0.001*.

### Requirements for caregivers

**Figure 4:**
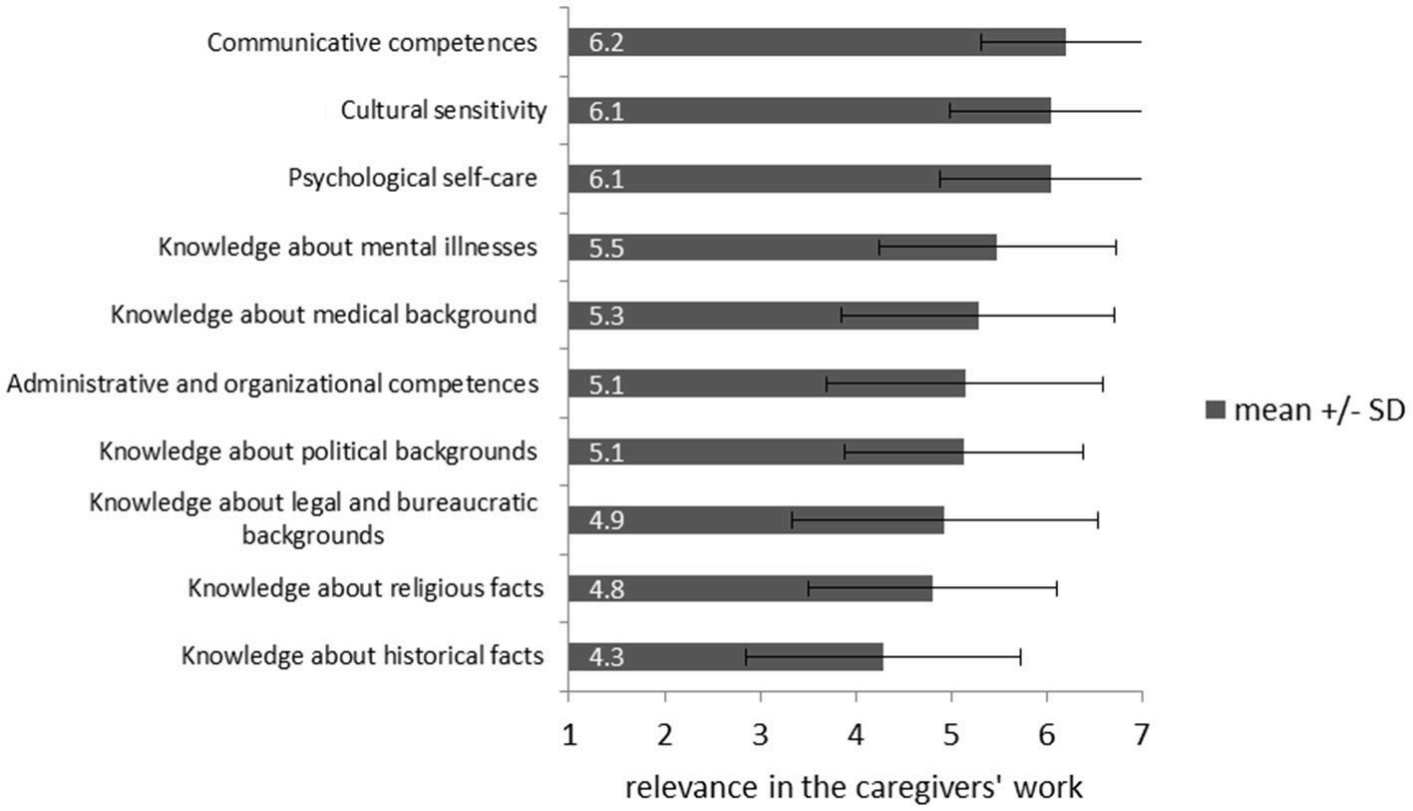
Rating of requirements for caregivers working with IS-victims from 1 (“irrelevant”) to 7 (“very relevant”).

As highly significant for the work in the HAP the participating caregivers rated “*Communicative competence”* (*M* = 6.20, *SD* = 0.89), “*cultural sensitivity”* (*M* = 6.05, *SD* = 1.07), and “*psychological self-care”* (*M* = 6.05, *SD* = 1.17) were rated as highly significant for the work in the HAP (Figure 4).

## Discussion

This is the first study that investigated the prevalence of secondary traumatization and its determinants in caregivers working with IS-traumatized women and children. The study showed that working with patients who suffered from torture (physical, mental, and sexual violence) while in the captivity of a radical terror organization can lead to trauma-specific symptoms at caregivers' side. In 22.9% of the caregivers secondary traumatization as defined by the Questionnaire of Secondary Traumatization was present at least once throughout their work in refugee-care, with 8.6% of participants indicating severe secondary traumatization. Since these professional lifetime prevalence rates occurred during a short time period of an average of 16.3 months, they demonstrate a substantial mental health risk to caregivers working with traumatized refugees. This is an important finding since secondary traumatization not only affects the well-being of the caregiver but may also impact the care they are providing. Disrupted alliances between caregivers and clients, violations of professional boundaries, or inadequate reactions to the client's trauma could be the consequence (40).

FST-scores found in this study are comparable to those of interpreters working in common refugee-care (19) and even almost as high as the professional lifetime-prevalence of secondary traumatization in trauma-therapists (17, 18). Clinicians with higher levels of exposure to victims of sexual violence are statistically more likely to exhibit trauma symptoms themselves (41). Since the beneficiaries of the HAP were especially selected for the program due to their extremely traumatic experiences in IS-captivity, where sexual violence was used as a weapon of war (30), HAP-caregivers are working in a context which puts them at high risk for developing secondary traumatization. This corresponds with the presented data.

### Distressing factors of caregivers

As the most important distressing factors for caregivers this study detected witnessing the suffering of the refugees by the caregivers, cultural differences in child-rearing between the refugees and the caregivers and the current humanitarian developments in Northern Iraq. These main distressing factors show how diverse the requirements for refugee-caregivers are. In their everyday work, they are not only confronted with highly traumatized clients but also with cultural differences and ongoing humanitarian catastrophes in the home countries.

Associated with secondary traumatization in caregivers is their psychological stress resulting from witnessing the refugees suffer, from the reports of the traumatic experiences of the beneficiaries and from witnessing beneficiaries contacting their offenders in Northern Iraq. Furthermore, stress resulting from the intermixture of private and professional aspects, from excessive expectations of beneficiaries regarding the caregivers' profession or role and from autonomy/dependency conflicts between caregivers and beneficiaries correlates with secondary traumatization. Regarding cultural differences, only stress due to differences in gender roles is associated with secondary traumatization. Concrete implications of these results are summarized in **Figure 6**.

**Figure 5:**
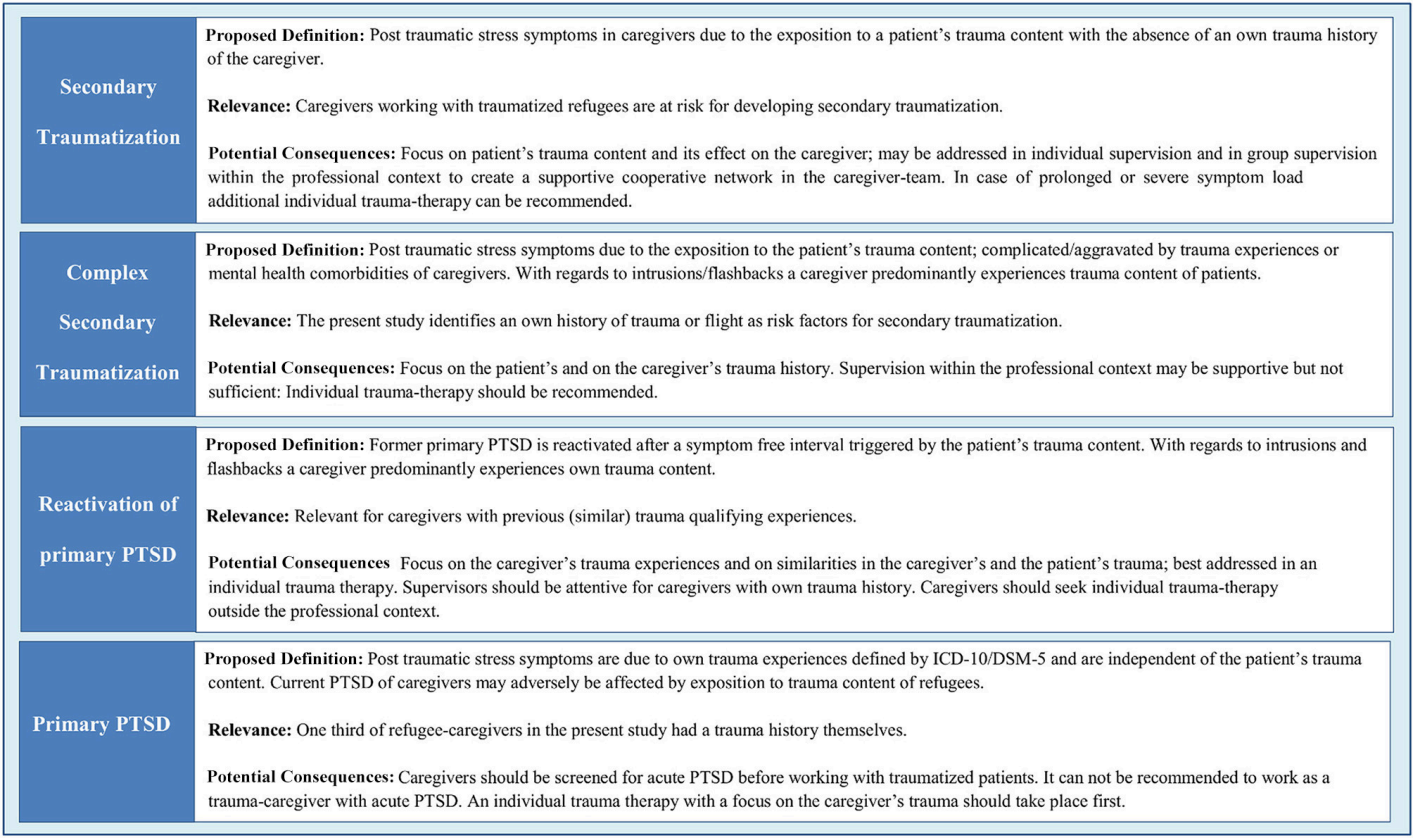
Proposed classification of trauma-associated psychological burdens of caregivers working with traumatized refugees.

As a main finding of this study, a personal history of traumatic experiences could be identified as a risk factor for secondary traumatization. In former studies, it has been shown that previous experience of significant stressors increases current levels of distress, beyond that which is accounted for by present stressors (42). This study not only supports this finding but widens it by applying it to symptoms of secondary traumatization, suggesting that a personal trauma history makes caregivers more vulnerable to the trauma stories of others. These results are consistent with other studies finding an association between personal history of trauma and secondary traumatization (19, 20, 43, 44). However, it has been suggested that being exposed to details of a patient's trauma might trigger one's own similar traumatic experiences. If this is the case, then the occurring symptoms could be due to the primary traumatization rather than symptoms of secondary traumatization (23, 45, 46). It has also been critically discussed whether the association between own trauma experience and secondary traumatization is due to a lack of construct validity of instruments which can lead to a lack of distinction between actual PTSD, reactivation of PTSD-symptoms, and secondary traumatization (45, 46). However, unlike many former studies that used common PTSD-questionnaires to assess secondary traumatization, this study avoided this critique by using a reliable and valid secondary traumatization questionnaire with good internal consistencies. The FST was specifically designed and evaluated for measuring trauma symptoms that are uniquely associated with the client's and not the caregivers personal traumatic experience (17, 34). Therefore, the present study aimed to measure distinct secondary traumatization load differentiated from actual PTSD-symptoms in caregivers or a reactivation of former PTSD-symptoms. Furthermore, this study found that caregivers with own trauma histories show more secondary traumatization than caregivers without own trauma histories. These findings argue for another differentiation between common secondary traumatization and secondary traumatization aggravated by own trauma experiences. Based on theoretical considerations and the findings of this study, four categories of trauma-associated psychological burdens and their practical implications can be hypothesized (Figure 5). In further studies, the kinds of trauma caregivers have already experienced themselves should be assessed further to test if the exact type of trauma influences the vulnerability for secondary traumatization.

In the study sample, one-third of the caregivers (24.1%) already experienced at least one traumatic event in their lives themselves. This lifetime-prevalence rate is comparable to the rate of 23.8% for traumatic experiences found in the general population in Germany (47). Nevertheless, there are also references that people working in refugee-care are even more likely to have experienced a traumatic event themselves than the general population (48). Therefore, an own trauma history is a highly relevant risk factor for secondary traumatization in refugee-caregivers.

More specifically, this study identified an own history of flight as a relevant risk factor for secondary traumatization in refugee-caregivers. In this case, the caregiver's personal experiences show similarities to the refugees' life stories. In qualitative interviews, trauma therapists already noticed that similarities to their own life could be the reason for a lack of necessary professional distance which could lead to secondary traumatization (17). The findings of this study seem to support this assumption. Since interpreters often come from migrant or refugee families, this might be one explanation for the high percentage of interpreters suffering from secondary traumatization.

The results of this study also show that a high frequency and duration of direct contact with refugees is another risk factor for secondary traumatization. This finding is in line with other studies where caregivers with more exposure to traumatized patients reported higher levels of post-traumatic stress symptoms (15, 41, 49, 50). In addition, the number of children the caregivers worked with correlated significantly and positively with FST-scores whereas the number of women cared for was not significantly associated with secondary traumatization. This finding indicates a difference between the work with IS-traumatized children and the work with IS-traumatized women regarding secondary traumatization. Hence, future studies comparing secondary traumatization in caregivers exclusively working with children compared to adult settings could be beneficial.

A preoccupied attachment style, characterized by an over-involvement in close relationships in combination with a low self-esteem, can also increase the risk for secondary traumatization. Since Kindermann et al. identified a preoccupied attachment style as a resilience factor for secondary traumatization in interpreters working in refugee-care (19), we hypothesized the same for this study sample. However, as the results show, a preoccupied attachment style does not only fail to prevent secondary traumatization in refugee-caregivers, but qualifies as a potential risk factor for secondary traumatization. In contrast, fearful and dismissing attachment styles, both characterized by a negative perception of others, did not significantly explain variance of the symptom load. These results suggest that only attachment styles based on a positive perception of others (preoccupied and secure) can serve as potential determinants of secondary traumatization.

### Resources of caregivers

The main resources of caregivers are talking to colleagues from their care centers, the awareness of doing something meaningful, and the already acquired knowledge and competence. Considering the communication with colleagues as helpful is associated with lower secondary traumatization scores. This applies to the communication with colleagues from the same care center and from other care centers. Since there is no significant difference in secondary traumatization symptom load between caregivers with and without intervision, it is possible that the communication with colleagues supports caregivers especially in an informal, unstructured way.

As another main finding, a secure attachment style could be identified as a potential resilience factor for secondary traumatization. This finding goes in line with previous research (19) and with the detected resources in the present study such as communication with colleagues. Since caregivers with secure attachment styles, defined as being comfortable with intimacy and autonomy (27), are more likely to have functioning relationships (51), the results of this study point out the importance of a reliable social network in preventing secondary traumatization, especially but not exclusively with coworkers.

Although supervision is strongly recommended in the recent literature in order to prevent secondary traumatization (52), it could not be identified as a significant resilience factor for secondary traumatization in the present study. However, 71.6% of caregivers rated their need for supervision as “rather high” to “very high” and supervision was rated as “rather helpful” on average. This finding suggests that even if the supervision received in the HAP does not significantly prevent secondary traumatization, caregivers wish for, and benefit from it to reduce the burden of work related stress. Furthermore, the primary goal of supervision is not only to prevent secondary traumatization but improve other aspects of professional well-being and indeed the quality of care delivered. Moreover, the specific effects of supervision may only be sufficiently investigated in controlled prospective fashion when quantity and quality of supervision are also considered.

**Figure 6:**
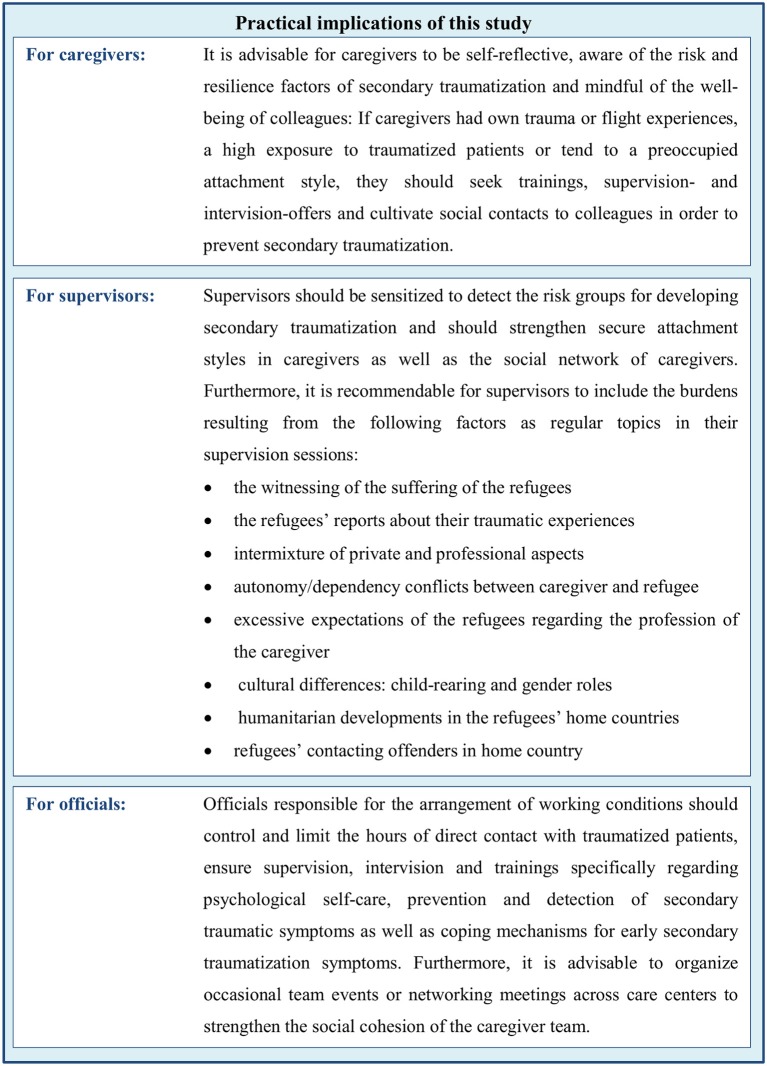
Specific recommendations for caregivers, supervisors, and officials in refugee-care based on the results of this study to prevent secondary traumatization and work-related stress.

Since the already acquired knowledge and competence are considered a main resource of caregivers, seminars before, during and after working in refugee-care could prevent work-related psychological strain. Although the importance of trainings for caregivers is acknowledged in recent literature (53), no significant differences in the severity of secondary traumatization emerged between caregivers with and without further relevant trainings. The results of this study suggest a more project-orientated offer of seminars where communicative competences, cultural sensitivity, and psychological self-care are trained as important competences in the work with refugees. Such professional trainings should be developed, standardized and evaluated in future research projects to guarantee a benefit for caregivers, especially for potentially vulnerable groups as identified in this study. For specific recommendation regarding prevention of secondary traumatization based on the results of this study see Figure 6.

## Limitations

This study shows several limitations: First, the small sample needs to be noted as a restriction of the present study. Moreover, since the networking meeting was a voluntarily event a selection bias due to taking part by more involved or dedicated caregivers may be present. However, it is hardly realistic to recruit larger samples of caregivers that work exclusively with IS-victims. Given the small number of HAP-caregivers in total and the high response rate in this study, the generalization of the study results for caregivers in the context of the HAP can be assumed. Second, the heterogeneity regarding different professions in the study sample with social workers as the predominant subgroup could be seen as a restricting factor for generalizing the findings for one or the other of the individual professions. For a closer look at the different professions in refugee-care and interprofessional differences regarding secondary traumatization beyond descriptive data, future research should consult larger subgroups of the relevant professions. Third, since secondary traumatization symptoms were captured retrospectively, the reported data underlies the validity-limitations of retrospective data collection. Forth, the cross-sectional study design limits the generalizability of the results and hinders inference of causal relationships. The role of the detected potential risk and resilience factors should therefore be considered as hypothetical and needs to be confirmed by longitudinal data.

## Conclusion

This is the first study to explore secondary traumatic symptoms, burdens and resources in caregivers working with women and children who suffered from extreme IS-violence in the form of torture, slavery, and sexual violence by using inter alia an instrument specifically designed and evaluated to detect secondary traumatization. The findings show that caregivers working in this context of refugee-care are a vulnerable group for developing secondary traumatization. The results of the present study also indicate that secondary traumatization varies by both individual characteristics such as attachment styles and personal experiences, as well as by environmental characteristics such as the dose of exposure to traumatized patients. The results of this study have implications for the selection, training, and continuing education and organizational support of caregivers working with traumatized refugees especially when extreme forms of traumatic content such as IS-violence against women are present in the respective clients.

## Ethics statement

This study was carried out in accordance with the recommendations of the ICH-GCP-guidelines, Declaration of Helsinki. The protocol was approved by the ethics committee of the medical faculty of the University of Tübingen and the University Hospital Tübingen named Ethik-Kommission an der Medizinischen Fakultät der Eberhard-Karls-Universität und am Universitätsklinikum Tübingen. All subjects gave written informed consent in accordance with the Declaration of Helsinki.

## Data availability statement

The raw data supporting the conclusions of this manuscript will be made available by the authors, without undue reservation, to any qualified researcher.

## Author contributions

JD, CR-O, PW, and FJ planned and conducted the study with the support of MB and HS. JD analyzed the data and wrote the manuscript with support from FJ. CN, SZ, JH, JK, NA, PP, and NG supported the study and the writing process with ideas and feedback.

### Conflict of interest statement

The authors declare that the research was conducted in the absence of any commercial or financial relationships that could be construed as a potential conflict of interest.
